# A prospective cohort study of depression in pregnancy, prevalence and risk factors in a multi-ethnic population

**DOI:** 10.1186/s12884-014-0420-0

**Published:** 2015-01-24

**Authors:** Nilam Shakeel, Malin Eberhard-Gran, Line Sletner, Kari Slinning, Egil W Martinsen, Ingar Holme, Anne Karen Jenum

**Affiliations:** University of Oslo Institue for health and society, departement of general practice, Norway, Avdeling for allmennmedisin, institutt for helse og samfunn, Universitetet i Oslo, Norge, Postboks 1130 Blindern, Oslo, 0318 Norway; Department of Psychosomatics and Health Behaviour, Division of Mental Health, Norwegian Institute of Public Health, Oslo, Norway; Health Services Research Center, Akershus University Hospital, Lørenskog, Norway; Institute of Clinical Medicine, Campus Ahus, University of Oslo, Lørenskog, Norway; Department of Child and Adolescents Medicine, Akershus University Hospital, Lørenskog, Norway; The Center for Child and Adolescent Mental Health, Eastern and Southern Norway (RBUP), Oslo, Norway; Department of psychology, University of Oslo, Oslo, Norway; Division of Mental Health and Addiction, Oslo University Hospital, Institute of Clinical Medicine, University of Oslo, Oslo, Norway; Oslo University Hospital Ullevål, departement of Biostatic, Epidemiology and Health economics, Oslo, Norway; Department of General Practice, Institute of Health and Society, Faculty of Medicine, University of Oslo, Oslo, Norway; Faculty of Health Sciences, Oslo and Akershus University College of Applied Sciences, Oslo, Norway

**Keywords:** Depression, Mental health, Risk factors, EPDS, Pregnancy, Ethnic groups

## Abstract

**Background:**

Depression in pregnancy increases the risk of complications for mother and child. Few studies are done in ethnic minorities. We wanted to identify the prevalence of depression in pregnancy and associations with ethnicity and other risk factors.

**Method:**

Population-based, prospective cohort of 749 pregnant women (59% ethnic minorities) attending primary antenatal care during early pregnancy in Oslo between 2008 and 2010. Questionnaires covering demographics, health problems and psychosocial factors were collected through interviews. Depression in pregnancy was defined as a sum score ≥ 10 by the Edinburgh Postnatal Depression Scale (EPDS) at gestational week 28.

**Results:**

The crude prevalence of depression was; Western Europeans: 8.6% (95% CI: 5.45-11.75), Middle Easterners: 19.5% (12.19-26.81), South Asians: 17.5% (12.08-22.92), and other groups: 11.3% (6.09-16.51). Median EPDS score was 6 in Middle Easterners and 3 in all other groups.

Middle Easterners (OR = 2.81; 95% CI (1.29-6.15)) and South Asians (2.72 (1.35-5.48)) had significantly higher risk for depression than other minorities and Western Europeans in logistic regression models. When adjusting for socioeconomic position and family structure, the ORs were reduced by 16-18% (OR = 2.44 (1.07-5.57) and 2.25 (1.07-4.72). Other significant risk factors were the number of recent adverse life events, self-reported history of depression and poor subjective health three months before conception.

**Conclusion:**

The prevalence of depression in pregnancy was higher in ethnic minorities from the Middle East and South Asia. The increased risk persisted after adjustment for risk factors.

## Background

Depression in pregnancy shares a similar symptom profile as depression occurring in other contexts, involving changes in appetite, feelings of guilt and low energy [1]. It may also disrupt the foetal developmental process [2] and increase the risk of adverse health outcomes for the mother and foetus such as preeclampsia and preterm birth [3]. Substance and alcohol abuse and cigarette smoking are associated with depression, and may further increase the risk of pregnancy complications [4,5]. The strongest risk factors appear to be a history of anxiety and depression [6,7], adverse life events [8] and lack of support, from the partner and others [3]. Depression in pregnancy may persist into the postpartum period [9] and disrupt the parenting behaviour, the attachment process between the mother and baby, as well as the relationship with the partner and any other children [10]. Although studies from pregnancy are few, they indicate that depression during pregnancy is as prevalent as during the postpartum period [11].

Ethnic minorities in Western countries are often exposed to stressors both before and during migration and after resettlement, such as lack of social integration, language problems, exposure to racism, unhealthy nutrition and poor housing conditions. These factors may adversely affect their mental health [12,13], placing ethnic minorities at higher risk for the development of postpartum depression [14]. Another aspect is the potential tension that immigrants from some countries with cultures more dominated by collective values may experience when exposed to the more individualistic Western societies. When there is a congruency between own preferences and those in the area of residence, living in neighbourhoods with many others from the same culture may be protective compared to living relatively alone in an affluent districts [15,16]. However, for an immigrant who does not share the collective values of his or her group, living among more traditionally oriented family members or neighbours may represent an extra burden due to social control mechanisms. Further, the care of women during pregnancy and postpartum differ in Europe compared to Asia and Africa [17]. Not living close to their extended family, and not having access to the type of care they are used to in their home countries can be distressing and negatively affect their mental health. However, little is known about depressive symptoms and risk factors for depression in pregnancy for ethnic minority groups living in Western societies [18-24].

Hence, the primary aim of this study was to determine the prevalence of depression in pregnancy and its associations with ethnicity and other risk factors. In addition, for immigrant groups we aimed to assess the importance of factors related to the level of social integration into the Norwegian mainstream society.

## Methods

### Design, study population and setting

This study is part of the STORK Groruddalen Research Program, with the primary goal to reduce short and long-term health risks for mothers and offspring by initiating knowledge-based and culturally-sensitive interventions among pregnant women and their families. Data are drawn from a population-based, prospective cohort of 823 healthy pregnant women attending the Child Health Clinics (CHC) for antenatal care in three administrative districts in Groruddalen, Oslo, covering a population of 82 500, between 2008 and 2010 [25]. Groruddalen was chosen as it covers affluent as well as more deprived residential areas, has a population with a diverse socioeconomic status and a high proportion of ethnic minorities. The majority (75-85%) of pregnant women residing in this area attend the CHC for antenatal care [25]. Antenatal care of normal pregnancies in Norway is carried out in primary care, either at the public CHC alone, in combination with the general practitioner (GP), or by the GP alone. All information material and questionnaires were translated to Arabic, English, Sorani, Somali, Tamil, Turkish, Urdu and Vietnamese and quality controlled by bilingual health professionals. The participation rate was 74% (range 64-83% among ethnic groups), and the participating women were found representative of all pregnant women attending the CHCs [25,26]. Women were eligible if they: 1) lived in the study districts; 2) planned to give birth at one of two study hospitals; 3) were at <20 weeks of gestation at inclusion; 4) could communicate in Norwegian or any of the other eight specified languages; and 5) were able to give written consent.

Women with diseases necessitating intensive hospital follow-up during pregnancy were excluded. Data were collected at study inclusion (mean gestational week (GW): 15.1 (SD: 3.4)), at GW 28 (mean GW: 28.3 (1.3)) and in the postpartum period *(mean number of weeks postpartum: 14.3. (2.8))*. The STORK Groruddalen project has been described in detail previously [25,26]. In short, questionnaire data covering demographics, health issues and psychosocial factors were collected through interviews by midwives at all three visits, assisted by professional translators when needed. Staff members were certified after extensive training to ensure standardized data collection procedures.

The study was approved by The Regional Ethics Committee South-East and the Norwegian Data Inspectorate, (reference number: 2007.894). All participants gave their written informed consent prior to their inclusion in the study.

### Primary outcome variable – depression in pregnancy

We used The Edinburgh Postnatal Depression Scale (EPDS) [27], a 10-item, self-rating scale, originally designed to identify women at risk for postpartum depression, but also later used for depression in pregnancy [28,29]. The EPDS has previously been thoroughly validated for use in postnatal, non-postnatal [30] as well as in pregnant women [31-34]. This measure is now the most frequently used screening instrument in epidemiological studies related to depression in pregnancy and postpartum [35]. The EPDS sum score ranges from 0 to 30 points, with higher score indicating more symptoms. This instrument has been found to have good sensitivity and specificity when tested against other assessment methods [36]. High test-retest reliability (0.81) has also been demonstrated in other pregnant populations [31]. The EPDS has previously been used for pregnant women in numerous Norwegian studies [11,37-45].

We used the validated Norwegian translation, which has satisfactory psychometric properties among postnatal Norwegian women, with satisfactory measures of reliability, including a Cronbach’s alpha of 0.81 and a test-retest correlation of 0.74 [36,46], and the official translations of seven other languages (Arabic, English, Somali, Tamil, Turkish, Urdu and Vietnamese) at the visit in GW 28 [27,47]. The Sorani version was translated by the City Services Department, The Interpreting Service in Oslo, and quality-checked for clarity and content validity by bilingual health professionals. In accordance with most epidemiological studies, we used the proportion scoring above a cut-off EPDS score ≥10 as a proxy measure for depression [48], both when referring to the prevalence data and in the subsequent logistic regression analyses. We also performed supplementary sensitivity analyses based upon an EPDS total score ≥ 12.

### Exposure variables

#### Ethnicity

Ethnicity was considered as our main exposure variable, and was defined according to each participant’s country of birth or the participant’s mother’s country of birth if the participant’s mother was born outside of Europe or North America [26]. The Western European group comprised participants born in Norway (93%), Sweden, Denmark, other Western European countries and North America. Women with origin from Eastern Europe, Africa, Asia, South - and Central America are referred to as ethnic minorities, further categorized as Middle Easterners (mainly from Iraq, Iran and Turkey), South Asians (mainly from Pakistan and Sri Lanka), and others (from Eastern Europe, Africa South of Sahara, East Asia and South and Central America) [26].

#### Adverse life events

Seven questions assessed stressful life events: 1) serious illness or injury (self), 2) serious illness or injury of close family member, 3) death of close family members, 4) divorce or separation from a long-term relationship, 5) unemployment or serious problems finding a job, 6) major concerns for children and 7) any other major event not already covered, like serious financial problems, difficulties at work, serious conflict etc. Response options were “*yes”* or “*no*”. Women reported adverse life events at inclusion, covering any event occurring during the past six months, and at GW 28, covering events after inclusion. First, the number of events for each time period was calculated, and then combined to a single variable to reflect the total number of events from six months prior to the study inclusion up to GW 28. This variable was recoded as 0-1 event (reference), 2 events or ≥ 3events, driven by the distribution of data.

#### Subjective health

At inclusion, women were asked to self-report their subjective health during the last three months before pregnancy. The response categories were *“bad”, “not that good”, “good*” and “*very good*” [26], and responses were further dichotomized as “*bad”* or “*good”*.

#### History of depression

We used the lifetime major depression scale based on the DSM-IV criteria to assess history of depression [49]. This scale consists of five questions concerning sadness, appetite changes, and lack of energy, self-blame and concentration problems, with the response categories “*yes”* or “*no”.* Prior depression was defined as having had at least three simultaneously co-occurring symptoms with duration of at least two weeks. This information was collected at the postpartum visit, as it was recognized as an important risk factor for postpartum depression. Although the number of participants was somewhat lower at postpartum (N = 662), this variable was included due to its relevance for the outcome.

#### Other variables

Age, GW at inclusion, parity, level of education and occupational status were regarded as possible cofactors/confounding factors. Single parenthood (living without a partner) was treated as a potential risk factor (lack of emotional and practical support). Information about family structure (three generational households – living with own parents or parents-in-law) were also available. Maternal individual and household socioeconomic factors used in the analyses were maternal educational level, pre-pregnancy employment status, household crowding (persons per room) and housing tenure (owning vs. renting). We also explored factors related to the level of social integration for immigrant groups into the Norwegian mainstream society, here represented by variables reflecting language skills (three different questions), time of residency, social interaction with ethnic Norwegians and use of Norwegian media. Ethnic Norwegian participants were not asked these questions but were automatically given the highest value for these variables.

### Study sample

**Figure 1:**
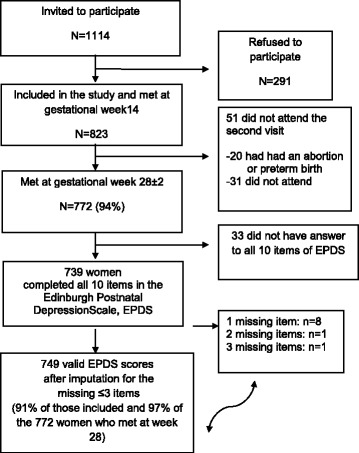
**Flowchart of study sample selection.**

From the total cohort of 823 women included in the study, 772 attended the follow-up at GW 28. 739 women had complete valid scores for all EPDS items at week 28. An additional 10 women had ≤ 3 missing items, for which mean values were imputed leaving a study sample of 749 women (Figure 1).

### Statistical methods

Descriptive statistics are provided as mean (SD), median (interquartile range; IQR) and frequency (%) as appropriate. To compare groups, chi-square tests were used for categorical data and T-tests or Mann-Whitney U for continuous variables as appropriate, depending on their distribution.

Most variables reflecting socioeconomic position (SEP) and level of integration are strongly correlated and interact with each other. However, they clearly represent different dimensions of societal and contextual factors. We therefore aimed at reducing dimensionality by extracting the smallest number of components that accounted for most of the variation in the original multivariate data. To achieve this goal, all individual and household markers of maternal SEP and variables related to the level of integration were entered into a principal components analysis (PCA) [50]. Two components were extracted. The first component was highly correlated with the predefined markers of the level of integration and was skewed to the right, reflecting that the ethnic Norwegians all had high scores. The second component had high correlations with the predefined individual and household markers of SEP. This score was normally distributed. Maternal occupational class was equally correlated with both components. Single parenthood was not correlated with other markers of SEP or the level of integration and was therefore not included in the PCA. The PCA scores were further dichotomized, into the 40% with the lowest scores versus the 60% with highest scores for SEP and level of integration, as the score values may be difficult to interpret directly from the tables. We examined different cut-off values (20/80 and also three categories with different cut-off values), but found that the pragmatic 40/60 cut point had a sufficient number in the lowest category, and still carried important information. We used both the PCA scores and the individual components in the analyses.

Using univariate and multiple logistic regression analyses, a directed acyclic graph was drawn and an etiological approach was chosen to assess the association between the explanatory factors and depression in pregnancy. In a timeline, ethnic origin was considered as a point of departure, followed by SEP and then depression before pregnancy. Single parenthood, subjective health prior to pregnancy and the sum score of recent adverse life events were considered as risk factors, whereas age, parity and the pregnancy stage (gestational week at inclusion) were included as potential confounders. Level of social integration was considered as an intermediate factor. Our primary research question was whether depression was associated with ethnic origin. The second question was whether the level of social integration was associated with depression in ethnic minority women. To address these aims, univariate and multiple logistic regression analyses were performed with depression (EPDS score ≥10; yes/no) as the dependent variable. Two models were fitted to address the primary research question. Model 1 used ethnicity as the main independent (exposure) variable and the covariates included gestational week at inclusion, single parenthood, adverse life events, subjective health three months before pregnancy and a history of depression. In model 2, SEP and family structure were added to the model.

To answer the second research question, ethnicity was replaced by level of integration, as over-adjustment was expected when including a variable highly correlated with ethnicity and falling on the pathway between ethnicity and depression in pregnancy. After removal of ethnicity from Model 2, we first used the PCA variable for level of integration. Then we assessed different indicators of social integration in the total sample, and then restricted analyses to the ethnic minority group. We used all available individual items thought to reflect level of integration, plus family structure, thereafter including SEP in all models. The model with the PCA variable reflecting the overall level of integration instead of the individual items had the best fit (Model 3).

All models were tested for interactions, entering cross-product terms one-by-one. The only significant interaction found was between ethnicity and age (p = 0.04), and age was therefore excluded from the final models as it had negligible impact on the estimates. Lastly, a sensitivity analysis was performed (Model 1-2 repeated) using EPDS sum score ≥12, for Western versus ethnic minority women. In these analyses, all minority groups were merged due to low numbers in some groups.

The statistical significance level was set to p <0.05. We report explained variance for the Models as Nagelkerke R-square (R^2^). The SPSS version 19 (IBM SPSS statistics, NY, USA) was used for all analyses.

## Results

### Characteristics of study sample

Of the 749 women with valid EPDS data, 444 (59.3%) were ethnic minorities. Baseline socio-demographic characteristics according to ethnic group are presented in Table 1. The ethnic minority women were younger, had less education, and higher parity and unemployment compared to Western European women. Heterogeneity was found across ethnic minority subgroups. No significant baseline differences were found between participants and the 74 excluded women on the following variables: ethnicity, age, GW at inclusion, parity, single parenthood, employment status and education.

**Table 1:** **Baseline characteristics for Western and ethnic minority women**

	**Total n =749**	**Western Europe n = 305**	**Ethnic Minority Group n = 444**	**South Asia n = 189**	**Middle East n = 113**	**Other Minorities n = 142**
	**n (%)**	**n (%)**	**n (%)**	**n (%)**	**n (%)**	**n(%)**
Age, mean (SD)	29.9 (4.84)	30.9 (4.50)	29.2 (4.93)	28.7 (4.46)	29.6 (5.46)	29.4 (5.07)
Week of inclusion, mean (SD)	15.1 (3.39)	14.2 (2.25)	15.7 (3.87)	15.6 (3.86)	15.1 (3.27)	16.2 (4.26)
Nulliparous	344 (45.9)	157 (51.5)	187 (42.1)	78 (41.3)	40 (35.4)	69 (48.6)
Single parenthood^1^	40 (5.3)	13 (4.3)	27 (6.1)	2 (1.1)	5 (4.4)	20 (14.1)
Education						
<10 years of education	120 (16.1)	9 (3.0)	111 (25.2)	34 (18.1)	42 (37.5)	35 (24.8)
10-12 years	295 (39.7)	95 (31.4)	200 (45.4)	93 (49.5)	51 (45.5)	56 (39.7)
13-16 years	229 (30.8)	126 (41.6)	103 (23.4)	48 (25.5)	16 (14.3)	39 (27.7)
university/college	100(13.5)	73 (24.1)	27 (6.1)	13 (6.9)	3 (2.7)	11 (7.8)
Unemployed in pre-pregnancy	219 (29.5)	36 (11.9)	183 (41.7)	71 (38.0)	59 (52.2)	53 (38.1)
Proportion with low score for socioeconomic position	294 (39.3)	61 (20)	233 (52.5)	88 (46.6)	72 (63.7)	73 (51.4)
Proportion with low score for social integration	300 (40.1)	17(5.6)	283 (63.7)	122 (64.6)	73 (64.6)	88 (62.0)
Self-reported adverse life events at inclusion^2^						
≥2 events	66 (9.1)	23 (7.8)	43 (10.0)	16 (8.6)	16 (14.5)	11 (8.2)
Self-reported adverse life events after inclusion^3^						
≥2 events	45 (6.1)	16 (5.4)	29 (6.5)	14 (7.4)	10 (8.8)	5 (3.6)
Poor subjective health 3 months before pregnancy	77 (10.4)	17 (5.6)	60 (13.7)	30 (16.0)	18 (16.2)	12 (8.7)
Previous history of depression^3^	114 (18.2)	61 (23.1)	53 (14.6)	21 (13.5)	15 (16.5)	17 (14.8)

^1^Living without a partner.

^2^Past six months.

^3^Data collected postpartum.

Values are n (%) if not stated otherwise.

### Prevalence of depression

**Figure 2:**
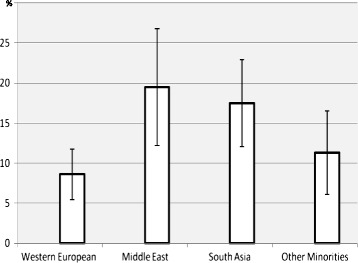
**Prevalence of depression in pregnancy in percentage, for the different ethnic groups with 95% confidence intervals, CI.**

A total of 97 women had an EPDS score ≥10, yielding a crude prevalence rate of 13.0% (95% CI: 10.59-15.41) for the whole study sample (Figure 2), 8.6% (5.45-11.75) for Western European women and 16.0% (12.59-19.41) for the ethnic minority group (p = 0.003). Women with Middle East ethnic origin had the highest prevalence of 19.5% (12.19-26.81), compared to 17.5% (12.08-22.92) among South Asians and 11.3% (6.09-16.51) in the group of other ethnic minorities (Eastern Europe, Africa South of Sahara, East Asia, and South and Central America). Middle Eastern women also had the highest median EPDS score of 6 (IQR: 2; 9) compared with South Asians: median of 3 (IQR 1; 7.5), Western Europeans: median of 3 (IQR: 1; 5), and other ethnic minorities: median of 3 (IQR 1; 7).

In the total study population, 60 women had an EPDS score ≥12 (8.0% (6.06-9.94), and the breakdown according to group was 4.6% (2.25-6.95) in Western Europeans versus 10.6% (7.56-13.24) in the merged ethnic minority group (p < 0.001).

### Risk factors for depression in pregnancy

Among women with *EPDS* ≥10, a total of 72.3 % had an ethnic minority background (Table 2). A higher number of women with *EPDS* ≥10 were unemployed, had a low SEP, reported single parenthood, more recent adverse life events and poor subjective health.

**Table 2:** **Sociodemographic characteristics for women with Edinburgh Postnatal Depression Scale score above and below cut-off 10**

	**Total 749 (100%)**	**EPDS ≥10, n = 97 (13%)**	**EPDS < 10, n = 652 (87%)**	**p-value** ^**4**^
Ethnic minority women	444 (59.3)	71 (73.2)	373 (57.2)	0.003
Age, mean (SD)	29.9 (4.84)	30 (5.17)	29.9 (4.79)	0.743
Nulliparous	344 (45.9)	39 (40.2)	305 (46.8)	0.226
Single parenthood^1^	40 (5.3)	11 (11.3)	29 (4.4)	0.005
Educational level				
<10 years education	120 (16.1)	19 (15.6)	101 (19.6)	0.721
10-12 yrs, secondary level	295 (39.7)	39 (39.6)	256 (39.8)	
up to 4 years of higher education	229 (30.8)	28 (28.9)	201 (31.1)	
University/college	100 (13.4)	11 (11.3)	89 (13.8)	
Unemployed/working disabled (pre-pregnant)	219 (29.5)	39 (40.2)	180 (27.9)	0.013
Proportion with low SEP score	294 (39.3)	48 (49.5)	246 (37.7)	0.027
Proportion with low score for social integration	300 (40.0)	47 (48.5)	253 (38.8)	0.070
Sum score of recent adverse life events^2^				
0 or 1 event	544 (76.2)	49 (54.4)	495 (79.3)	<0.001
2 events	107 (14.9)	17 (18.9)	90 (14.4)	
≥3events	63 (8.8)	24 (26.7)	39 (6.3)	
Poor subjective health 3 months before pregnancy	77 (10.4)	22 (23.4)	55 (8.5)	<0.001
History of depression^3^	114 (18.2)	29 (36.3)	85 (15.6)	<0.001

^1^Living without a partner.

^2^From six months before pregnancy to gestational week 28.

^3^Data collected postpartum.

^4^Chi-square tests (%) for categorical variables or t-test (SD) for age (yrs).

Values are in n (%) if not stated otherwise.

In the logistic regression analyses using EPDS ≥10 as the dependent variable (Models 1 and 2), women from the Middle East and South Asia had significantly higher ORs than Western European women (Table 3). Ethnic minority origin from the Middle East (OR 2.81; 95% CI: (1.29-6.15)) and South Asia (2.72; 95% CI: (1.35-5.48)) remained independent risk factors for depression when adjusted for single parenthood, gestational week of inclusion, adverse life events, poor subjective health and history of depression (Model 1, R^2^ 19.7%).

**Table 3:** **Odds Ratio (OR) for depression in pregnancy in univariate and multiple logistic regression analyses**

	**Unadjusted OR**			*Model 1 (R ^**2**^ **= 19.7%)**			*Model 2 (R ^**2**^ **= 20.7%)**			*Model 3 (R ^**2**^ **= 18.9%)**		
	**OR**	**95% CI**	**p-value**	**OR**	**95% CI**	**p-value**	**OR**	**95% CI**	**p-value**	**OR**	**95% CI**	**p-value**
Ethnic origin (Western Europe ref)												
Middle East	2.60	1.40-2.80	0.002	2.81	1.29-6.15	0.010	2.44	1.07-5.57	0.034			
South Asia	2.27	1.31-3.94	0.003	2.72	1.35-5.48	0.005	2.25	1.07-4.72	0.033			
Other minorities	1.36	0.71-2.63	0.356	1.24	0.51-3.04	0.638	1.16	0.46-2.88	0.759			
Gestational week at inclusion	1.03	0.97-1.10	0.380	0.99	0.92-1.08	0.878	0.99	0.91-1.08	0.863	0.99	0.91-1.08	0.781
Age	1.01	0.97-1.05	0.743									
Single parenthood^1^	2.75	1.33-5.71)	0.007	2.91	1.035-8.18	0.043	2.74	0.96-7.84	0.060	2.05	0.79-5.36	0.143
Adverse life events												
(0-1 ref)												
2	1.91	1.05-3.46	0.033	1.54	0.77-3.08	0.222	1.58	0.79-3.17	0.199	1.64	0.82-3.27	0.161
≥3	6.22	3.46-11.18	<0.001	4.97	2.45-10.08	<0.001	4.95	2.43-10.10	<0.001	4.83	2.40-9.70	<0.001
Poor self-reported health^2^												
good (ref)												
bad	3.28	1.89-5.70	<0.001	2.00	1.01-3.99	0.048	2.08	1.04-4.16	0.039	2.29	1.16-4.48	0.016
Mother’s socioeconomic position (SEP)												
highest 60% (ref)												
lowest 40%	1.62	0.10-2.65	0.028				1.30	0.73-2.31	0.370	1.68	0.98-2.90	0.061
Mother’s level of social integration												
highest 60% (ref)												
lowest 40%	1.48	0.97-2.28	0.072							1.84	1.07-3.17	0.027
Previous history of depression^3^	3.01	1.86-5.14	<0.001	2.77	1.51-5.06	0.001	2.83	1.53-5.20	0.001	2.61	1.44-4.73	0.002
Read Norwegian newspapers/watch TV												
Daily(ref)												
Weekly	0.85	0.087-8.32	0.889									
Seldom	1.12	0.42-3.01	0.828									
Never	2.94	1.0-8.67	0.051									
Living together with in-laws	1.41	0.55-3.60	0.472				2.41	0.86-6.75	0.096			

^1^Living without partner.

^2^3months before pregnancy.

^3^Collected postpartum.

^*^Model 1-3: multiple logistic regression analyses with adjusted OR for included variables.

In Model 2, with additional adjustment for SEP and other available variables potentially reflecting social support (living with in-laws or with own parents), the ORs of the Middle Easterners and South Asians were reduced by 16-18% (Middle Easterners: 2.44; 95% CI: (1.07-5.57); South Asians: 2.25; 95% CI: (1.07-4.72)), but still remained strong and significant, indicating less residual confounding in this model (R^2^ 20.7%). In both model 1 and 2, reporting ≥ 3 recent adverse life events (six months before inclusion), a history of depression and poor subjective health three months before conception were robust and significant risk factors. Single parenthood was borderline significant in the last model (p = 0.06). In model 2, living with in-laws seemed to increase the risk of depression (OR 2.41; 95% CI: (0.86-6.75)), but did not reach significance (p = 0.096). When replacing living with in-laws with living with one’s own parents, the effect of family structure was the opposite, indicating a trend towards protection, although not significant.

To investigate our secondary aim, we replaced ethnicity by the PCA variable for overall level of integration (Model 3), due to the strong correlation between markers of social integration and ethnicity. In the alternative modelling strategy, the variable reflecting social integration was significant (OR = 1.84; 95% CI: (1.07-3.17)), including history of depression, recent adverse life events and poor subjective health three months before conception, although the total explained variance in this model was slightly reduced (R^2^ 18.9%).

To examine which of the individual items related to social integration contributed most to the model, we performed separate analyses within the subgroup of ethnic minority women. The PCA variable for level of social integration in Model 3 was replaced sequentially by the following variables one-at-a-time: 1) self-reported abilities in Norwegian, 2) time of residence in Norway, 3) *how often during the last year the participant read Norwegian newspapers/* watched Norwegian TV, 4) was visited by an ethnic Norwegian. Only reading Norwegian newspapers/watching Norwegian TV “never” or “less than once a week” during the last year was independently associated with depression in pregnancy (results not shown).

In the sensitivity analyses using a stricter definition with EPDS sum score ≥ 12 as the primary dependent variable, results remained relatively unchanged. Specifically, risk of depression was independently associated with ethnic minority background (OR = 3.3, P = 0.007), self-reported history of depression and recent adverse life advents. With an EPDS cut-off value of ≥ 12, the R^2^ was 22.4% when using the same variables as in Model 2.

## Discussion

Our study adds to the sparse literature about depression in pregnancy among multi-ethnic populations. We found that depression in pregnancy (defined as an EPDS score ≥ 10), was twice as prevalent in ethnic minority women as Western Europeans. However, this increased risk did not apply equally to all groups of ethnic minority women. Middle Eastern and South Asian women had a significantly higher OR than the other minority women and Western Europeans, even after adjusting for known risk factors, including SEP. A history of depression, adverse life events and poor subjective health prior to pregnancy were independent risk factors, while single parenthood was borderline significant.

When replaced by ethnicity, level of social integration functioned as a significant risk factor for depression in pregnancy, in addition to a history of depression, adverse life events and poor subjective health before pregnancy. Specifically, results indicated that some aspects of social integration, such as reading Norwegian newspapers or watching Norwegian TV never or less than once a week, may serve as a proxy for factors related to integration that mediate the increased risk of depression observed in ethnic minority women.

Most previous studies have used an EPDS score ≥10 as a proxy for depression. In a Norwegian study of postpartum women to establish the validity of the EPDS against clinical diagnostic interviews and other screening instruments, an EPDS score of ≥10 had a sensitivity of 100% and specificity of 87% [46], compared to a sensitivity of 95% for major depression and a specificity of 93% for the English version [48]. However, false positives might be expected when using the EPDS as a screening instrument in clinical practice, thereby necessitating a clinical evaluation or diagnostic test to identify cases of clinical depression in need of treatment. In this regard, women with EPDS ≥10 are not necessarily clinically depressed, but have an increased level of depressive symptoms, which in turn implies a higher risk of depression. As our aim was to compare the symptom load in different ethnic groups in Europe mainly with Asian and African origin, living in the same residential area in the capital of Norway, we used the same cut-off value for all the pregnant women. Some researchers have employed a stricter EPDS score ≥12 [29], thus we also performed sensitivity analyses with this threshold. Results were essentially unchanged, with similar effect estimates, despite loss of power.

Prevalence estimates of depression in pregnancy differ substantially across studies, likely attributable to population heterogeneity, but also due to methodological differences such as choice of measurement [51,52]. Our findings are in accordance with a recent Cochrane meta-analysis of 21 studies [3], which reported that the prevalence of depression in pregnancy ranged from 7.4% in the first trimester to 12.8% in the second trimester, with a mean prevalence rate of 10.7%. Very few studies report results stratified by ethnicity, and this review failed to report the prevalence in minority groups. However, one study found that ethnic minority origin, specifically Asian, was associated with high EPDS scores [53]. A Swedish study found a significantly higher adjusted OR for depression in non-native speakers compared to women speaking the native language [24]*.* An Italian study investigating the prevalence of depression in pregnancy (EPDS ≥10) found a prevalence rate of 21.9% among the total sample, which included 7.5% of participants with a non-Italian background [54]*.* Women with depressive symptoms were more likely to be non-Italians and unemployed, with greater socioeconomic stress. Another Italian multi-centre, cross-sectional study similarly reported that a higher risk of depressive symptoms was associated with an ethnic minority background, a history of depression, being single and unemployment [55]*.* The Born in Bradford study of pregnant women used the General Health Questionnaire (GHQ-28), and found that ethnic minorities had higher risk of depression. Struggling financially, and for some ethnic groups not being part of the work force, also increased the risk of poor mental health [23]. While it is well-documented that a history of affective disorders is a strong risk factor for postpartum depression, only few articles have investigated the association between a history of depression and depression during pregnancy [3]. We found a strong association between a history of depression and depressive symptoms in pregnancy, regardless of ethnicity.

When comparing our results from pregnancy with studies using the EPDS instrument from countries where the ethnic minorities in Oslo originate from, they mostly report higher rates. We identified studies from Middle-East reporting prevalence rates from 19% to 57% [56-59]. Similarly, studies from Pakistan reported prevalence rates of depression to be 25% [60,61], but a recent study from Punjab, where most Pakistanis in Norway originally come from, reported even higher rates [62]. We did not find studies from the Tamil population in Sri-Lanka, only one for postpartum depression in India [63]*.* However, direct comparison is difficult due to methodological issues, not least the representativeness of included samples. Contextual factors also differ considerably.

Our study has several strengths. It is based upon a cohort design with a population-based sample including a high proportion of ethnic minorities, and the attendance rate was high. We have succeeded in including illiterate and recently immigrated women by adapting the study methods to the needs of these women [26]. The results are considered generalizable to the total cohort of 823 women included in the STORK-Groruddalen study, as no significant differences between participants and the those with missing data om EPDS (n = 74) were found.

We also have information on a broad range of socioeconomic measures, family structure, variables related to level of social integration, and we have applied an etiological approach. To our knowledge, only a few studies have explored depression in pregnancy among multi-ethnic populations and even fewer have presented analyses for several ethnic minority groups.

Some manifestations of pregnancy, particularly during the first trimester, may mirror symptoms of depression, such as fatigue, poor appetite and sleeping problems. As a consequence, depressed pregnant women often tend to have higher scores than non-pregnant women on available diagnostic tools, such as the Centre for Epidemiologic Studies Depression Scale (CES-D) [5]. The EPDS does not include any somatic symptoms, rendering this measure methodologically advantageous to use among pregnant women [64,65].

In our study, the EPDS was administered through interviews performed by certified study midwives. However, some women may have felt uncomfortable reporting problems in a face-to-face setting with a health care professional. If so, this may have led to underreporting and consequently an underestimate of the prevalence of depression. This may have been the case in a previous Norwegian study of postpartum depression in Pakistani women, in which the prevalence of depression was found to be 7.6% [66]. In that study, the women were interviewed at home, with family members, sometimes the husband, being present or acting as translators.

Nevertheless, there are some noteworthy limitations. We did not have direct measures of social support, but relied upon indirect measures like single parenthood and family structure. Further, information about history of depression was self-reported and collected in the postpartum period, thereby subject to various recall and memory biases associated with retrospective self-report. We cannot rule out that if a woman had her first depressive symptoms ever in the index pregnancy, she refers to this episode. However, it seems unlikely that this may be the case for many women, and that this category error would have a large effect on the estimates. The small number in some ethnic minority groups limited study power and the number of categories included in the regression analyses, especially for the sensitivity analyses using EPDS ≥12 as the cut-off score. Heterogeneity within the relatively broad ethnic groups probably exists, and our grouping strategy may have also masked important within-group differences.

Lastly, an important issue for any study of multi-ethnic populations is the cross-cultural validity of the instrument used. The expression of depression may differ according to cultural context [47]. The EPDS instrument has been used extensively worldwide, with established psychometric properties across different contexts and cultures. We used official and validated versions to the extent possible. The Urdu version of the EPDS has been used in Pakistan with good psychometric properties [67]. The Tamil version has been validated in a Tamil population in India with high specificity and sensitivity [68], the Turkish version has satisfactory validity when used in a Turkish sample [69]. The Arabic and Vietnamese versions have also been validated and found to be reliable [47]. Although we used the official translation for the Somalian language, we were unable to find any validation studies for this version. We did not have the resources to formally validate our Sorani version of the instrument.

As in the rest of Europe, there is an increasing number of ethnic minorities in Norway. The awareness in health policy is growing about the importance of adapting services for these groups to meet their needs according to legal standards of equity, but little is known about the actual quality of care. When planning services for the ethnic minority groups, knowledge about relevant risk factors are necessary to design preventive and management strategies. The EPDS measure is so far not recommended as a routine screening test for antenatal and postpartum depression, as more studies about the effect of such a procedure on patient important outcomes in the Norwegian context is requested.

## Conclusion

Although Middle Eastern and South Asian women were more likely to be depressed, a low SEP partly explained their increased risk. Irrespective of ethnicity, a history of depression, recent adverse life events and poor subjective health remained independent risk factors for depression. Data also suggest that a low level of social integration may mediate the increased risk observed in ethnic minorities. However, cultural factors or other unmeasured variables may likely contribute to risk status. In sum, our findings point to the potential for improving mental health among high-risk ethnic minority pregnant women by improving socioeconomic conditions and facilitating integration.

## Abbreviation

CHC: Child Health Clinics
GP: General practitioner
GW: Gestational week
SD: Standard deviation
EPDS: Edinburgh Postnatal Depression Scale
IQR: Interquartile range
SEP: Socioeconomic position
PCA: Principal component analysis
OR: Odds ratio
GHQ: General health questionnaire
CES-D: Center for epidemiologic studies scale
